# Endobiliary Photodynamic Therapy in Cholangiocarcinoma: Clinical Outcomes, Patient Selection, and Procedural Context

**DOI:** 10.3390/curroncol33060343

**Published:** 2026-06-09

**Authors:** Xuewu Zhang, An Jiang

**Affiliations:** Department of Hepatobiliary Pancreas and Liver Transplantation, The Second Affiliated Hospital of Xi’an Jiaotong University, Xi’an 710006, China; zhangxuewu@stu.xjtu.edu.cn

**Keywords:** cholangiocarcinoma, photodynamic therapy, biliary obstruction, biliary drainage, stent patency, narrative review

## Abstract

Endobiliary photodynamic therapy (PDT) in cholangiocarcinoma (CCA) is used mainly for local palliation of malignant biliary obstruction, particularly in extrahepatic and perihilar disease. This Review synthesizes the clinical evidence on endobiliary PDT while using drainage, infection control, stent strategy, light delivery, and systemic-therapy context as an interpretive framework rather than as practice recommendations. This narrative review was informed by targeted searching of PubMed/MEDLINE, Embase, and Web of Science from database inception through to 31 December 2025, supplemented by reference-list screening. We prioritized prospective studies, comparative cohorts, systematic reviews, and relevant guidance documents. Across the literature, the clearest support for PDT concerns selected local biliary palliation, including decompression, stent patency or delayed dysfunction, and symptom relief. Survival signals remain inconsistent: early positive studies contrast with the negative PHOTOSTENT-02 randomized trial and are highly confounded by drainage adequacy, infection control, retreatment strategy, and systemic-therapy access. We therefore interpret PDT as a context-dependent local biliary strategy rather than an established survival-prolonging treatment, and we highlight the clinical variables that make published outcome signals more or less interpretable.

## Simple Summary

Endobiliary photodynamic therapy (PDT) uses light-activated drugs delivered inside bile ducts to treat cholangiocarcinoma, a cancer of the bile duct lining. This review examines whether endobiliary PDT improves patient outcomes. The strongest evidence supports PDT as a palliative treatment that may help maintain bile duct drainage, reduce bilirubin levels, and delay stent blockage in selected patients with inoperable extrahepatic or perihilar tumors. However, evidence that PDT prolongs overall survival is inconsistent: early positive studies conflict with a later negative randomized trial, and results are complicated by differences in drainage adequacy, infection management, stent strategy, and systemic therapy access. We conclude that PDT should be understood as a context-dependent local palliative strategy rather than a proven survival treatment. Future studies should standardize procedural reporting and use biliary-specific endpoints alongside survival to better capture clinical value.

## 1. Introduction

Cholangiocarcinoma is often diagnosed when local biliary control and systemic disease management must be pursued simultaneously. Endobiliary photodynamic therapy (PDT) is a locoregional treatment in which a photosensitizer is administered and subsequently activated by intraductal light delivery, usually through endoscopic or percutaneous biliary access. The resulting photochemical reaction generates reactive oxygen species within the treated biliary segment [1,2,3,4,5]. In cholangiocarcinoma, the clinical rationale for endobiliary PDT is closely linked to palliation of malignant biliary obstruction, where local ductal control, drainage quality, stent patency, cholangitis risk, and continuity of systemic therapy may jointly influence patient outcomes.

Endobiliary PDT has therefore attracted interest because it can be delivered within the ductal lumen, paired with stenting and systemic therapy, and it is linked to plausible local photochemical effects, with broader vascular or immune consequences remaining less clinically defined. This Review is intended as a narrative clinical synthesis, not as a guideline or protocol statement. We focus on what current studies suggest about patient outcomes, where a local biliary role appears most plausible, and which procedural variables have the strongest effect on the interpretation of those outcomes [6,7,8,9,10,11,12,13].

## 2. Methods

This narrative review was informed by targeted searching of PubMed/MEDLINE, Embase, and Web of Science from database inception through to 31 December 2025, supplemented by reference-list screening. Search terms included combinations of cholangiocarcinoma, photodynamic therapy, endobiliary, endoscopic retrograde cholangiopancreatography, biliary stent, radiofrequency ablation, perihilar, distal, and intrahepatic. We prioritized prospective trials, comparative clinical studies, large observational cohorts, systematic reviews, and guideline or consensus documents for the clinical synthesis, while using mechanistic studies selectively for context. Supplementary materials provide additional context on registered biliary PDT trials, mechanistic/translational evidence, reporting items, and technical details relevant to endobiliary PDT interpretation. Because this was a narrative review, formal PRISMA screening and formal risk-of-bias grading were not performed; accordingly, translational concepts are discussed as exploratory and evidence-tiered rather than clinically established claims [1,2,3,4,5,6,7,8,9,10,11,12,13,14,15,16,17,18,19,20,21,22,23,24,25,26,27,28,29,30,31,32,33,34,35,36,37,38,39,40,41,42,43,44,45,46,47,48,49,50,51,52,53,54,55,56,57,58,59,60,61,62,63,64,65,66,67,68,69,70,71,72,73,74,75,76,77,78].

## 3. Clinical Context and Where Endobiliary PDT Fits

Endobiliary PDT is represented mainly in patients with malignant biliary obstruction from extrahepatic disease, especially perihilar cholangiocarcinoma (pCCA) and distal cholangiocarcinoma (dCCA), which are usually treated during endoscopic retrograde cholangiopancreatography (ERCP) and less often through percutaneous approaches. Read conservatively, the literature supports a narrow conclusion: in selected patients, PDT has been associated with more favorable local biliary outcomes, with signals of benefit most consistent for decompression, stent-related outcomes, and symptom control. By contrast, overall survival is difficult to interpret because both early positive and later negative studies are constrained by important uncertainty. Evidence in mass-forming intrahepatic cholangiocarcinoma (iCCA) is much thinner, so extrapolation from perihilar or distal extrahepatic obstruction to broader intrahepatic disease should remain restrained [6,7,8,10,11,12,13,15,16]. A more plausible use case appears to be obstruction-predominant extrahepatic or perihilar disease, whereas uncontrolled sepsis, inability to achieve interpretable drainage, rapidly progressive extra-biliary disease, or predominantly mass-forming iCCA materially weaken the case for endobiliary PDT.

### 3.1. Structured Synthesis of Clinical Outcomes

Does endobiliary PDT improve meaningful patient outcomes? The most defensible answer is narrow and qualified. Across randomized studies, comparative cohorts, and meta-analyses, the clearest support is for selected local biliary palliation in obstructing extrahepatic or perihilar disease. Signals for bilirubin improvement, stent patency or delayed stent dysfunction, and symptom or quality of life (QoL) benefit are more consistent than signals for overall survival (OS). However, even these biliary endpoints are difficult to compare across studies because the descriptions of drainage targets, stent strategy, cholangitis definitions, retreatment rules, and systemic-therapy exposure vary [2,10,11,12,13,20,21,22,23,24,27,28,29,30,31,32,33,34].

### 3.2. PDT Versus Endobiliary Radiofrequency Ablation (RFA) in Clinical Context

Comparisons between endobiliary PDT and endobiliary RFA remain context-dependent and inadequately standardized. PDT may be technically attractive in some long-segment or multifocal strictures, whereas RFA may be simpler in shorter focal lesions, but comparative effectiveness remains uncertain and cannot be reduced to a single anatomy-based rule [12,13,22,26,28].

### 3.3. Why Studies Disagree: A Clinical-Methodological Framework

For local biliary outcomes, the literature is somewhat more supportive, although still methodologically limited. Several studies describe improved biliary decompression, bilirubin response, delayed stent dysfunction, or reduced need for early reintervention after PDT-based treatment, although endpoint definitions and follow-up windows vary. Reported QoL or symptom benefits are limited but directionally supportive in some early and observational studies, whereas cholangitis and other infectious complications remain major competing risks [10,11,12,13,18,25,31,32,33,34]. The key clinical messages for interpreting endobiliary PDT are summarized in Table 1.

### 3.4. Practical Procedural Considerations That Affect Interpretation

**Figure 1:**
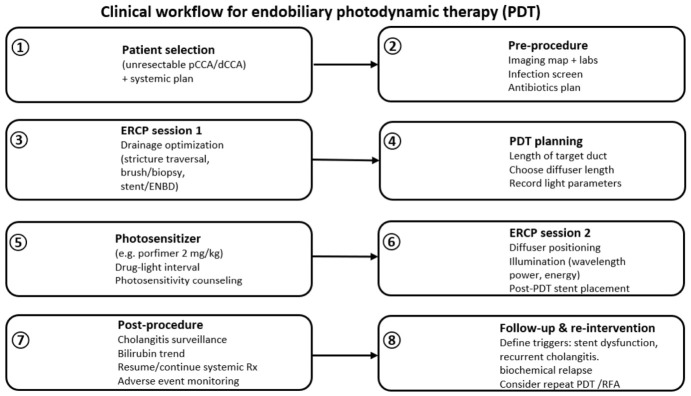
Clinical workflow for endobiliary photodynamic therapy (PDT) in cholangiocarcinoma. The workflow summarizes key clinical and procedural domains relevant to endobiliary PDT, including patient selection, pre-procedure assessment, ERCP-based drainage, PDT planning, photosensitizer administration, intraductal illumination, post-procedure care, and follow-up or re-intervention. Workflow elements are informed, where relevant, by ESGE and ASGE guidance on malignant hilar obstruction, ERCP adverse events, and antibiotic prophylaxis, but are intended as interpretive reporting domains rather than formal practice recommendations [17,18,19,25,29,30].

Procedural detail matters because local biliary outcomes can be obscured by differences in drainage adequacy, infection prevention, stent strategy, and delivery documentation. These variables should be understood as effect modifiers and reporting priorities, not as formal practice recommendations. Figure 1 summarizes a practical framework for interpreting endobiliary PDT studies, informed where relevant by ESGE and ASGE guidance [17,18,19,25,29,30].

Among these factors, cholangitis and incomplete drainage are especially important because they can dominate morbidity, trigger unplanned reintervention, and confound both patency and survival endpoints. Studies are more clinically readable when they specify drainage targets, antibiotic approach when incomplete drainage is anticipated, stent type and exchange strategy, and how post-procedure infections were defined and adjudicated [17,18,19,25,29,30].

When interpreting studies, readers should also consider whether post-PDT stent management was specified, whether self-expandable metal stents (SEMSs) or plastic stents were used appropriately for the setting, and whether rescue reintervention criteria were predefined [17,18,19,29].

#### Resource Use and Repeat Intervention Burden

From a practical standpoint, PDT may require additional procedure time, photosensitizer procurement and counseling, coordination of light-delivery equipment, and repeated ERCP for reassessment or retreatment. Formal cost-effectiveness data are limited, but procedure burden, healthcare use, and center-level availability should be considered part of the real-world treatment context rather than dismissed as implementation detail alone [11,18,25,33,34].

These issues do not negate a local palliative role, but they matter when judging whether a technically feasible strategy is also worthwhile for patients and health systems. The context-dependent comparison between endobiliary PDT and endobiliary RFA is summarized in Table 2.

**Table 2:** Context-dependent interpretation of endobiliary PDT and endobiliary RFA.

Domain	Endobiliary PDT (Typical)	Endobiliary RFA (Typical)
Mechanism	Photochemical injury with possible downstream vascular and immune consequences	Thermal coagulative necrosis via radiofrequency energy deposition
Energy–tissue interaction	Not governed by a classic heat-sink effect; depends on photosensitizer, oxygen, and light distribution	Thermal gradients; potentially influenced by perfusion/adjacent vessels (heat sink concept)
Strengths	Segmental duct coverage with cylindrical diffuser; repeatable local treatment; possible immunologic relevance	Procedure simplicity, immediate thermal debulking; widely available devices
Key limitations	Photosensitivity; requires photosensitizer logistics; light non-uniformity in complex hilar anatomy	Thermal injury risk; limited ablation length per application; unclear dose standardization
When PDT may be favored (hypothesis only)	Complex hilar strictures requiring longer segment light delivery; situations where a non-thermal approach is preferred	Short focal strictures where a limited ablation segment suffices, when photosensitizer logistics are prohibitive
Interpretive gaps	Need consistent reporting of photosensitizer, light delivery, retreatment, and stent strategy	Need consistent reporting of energy settings, catheter positioning, and clinically comparable endpoints

Note: Entries labeled as hypothesis reflect anatomy- and procedure-based interpretations of where PDT or RFA may fit in selected biliary scenarios; they are not evidence-based comparative recommendations. The major clinical outcome studies informing this interpretation are summarized in Table 3.

**Table 3:** Clinical outcome signals across major endobiliary PDT studies.

Study	Design/Sample Size	Population/Anatomic Context	Comparator/Key PDT Details/Follow-Up	Survival Signal	Biliary, QoL, and Safety Signal	Main Interpretive Limitation
Ortner et al., 2003 [10]	Randomized; *n* = 39	Nonresectable hilar/extrahepatic biliary obstruction	Stent alone; porfimer PDT + stent; follow-up NR	Median survival favored PDT + stent over stent-only treatment (493 vs. 98 days; *p* < 0.0001)	Reported improved biliary drainage and QoL	Small early-era RCT with early stopping
Pereira et al., 2018 (PHOTOSTENT-02) [11]	Multicenter randomized; *n* = 92	Advanced biliary tract cancer with biliary obstruction; mixed CCA/BTC population	Stent alone; porfimer sodium PDT + stent; median follow-up of 8.4 months	Direction did not favor PDT	No clear progression-free survival (PFS) or biliary advantage; higher overall grade (3–4) adverse-event burden and one treatment-related death	Mixed population and downstream-therapy imbalance, but important negative randomized signal
Cheon et al., 2012 [33]	Retrospective comparative; *n* = 72	Advanced perihilar cholangiocarcinoma with hilar obstruction	Biliary stenting alone vs. PDT + stent; photosensitizer/light-delivery details not consistently extractable from the available report; follow-up NR	Reported median survival favored PDT + stent over stent-only treatment (20.6 vs. 9.8 months; *p* < 0.001)	Reported longer survival and better local/stent-related outcomes; photosensitivity and cholangitis were clinically relevant adverse events, but event attribution was limited by retrospective design	Nonrandomized earlier-era cohort
Hong et al., 2014 [34]	Retrospective comparative; *n* = 74	Advanced perihilar cholangiocarcinoma	PDT alone vs. PDT + chemo; PDT delivered in a stent-managed hilar CCA context; follow-up NR	Median survival favored PDT + chemo over PDT alone (17.9 vs. 11.1 months; *p* = 0.05)	Suggests systemic-therapy context matters; not a direct test of PDT versus no PDT	Treatment-selection confounding
Li et al., 2020 [35]	Retrospective comparative; *n* = 62	Unresectable perihilar cholangiocarcinoma; ERCP/percutaneous cholangioscopy-directed treatment	Stent-only vs. hematoporphyrin PDT + stent; hematoporphyrin 2.0 mg/kg, 48 h drug-light interval, 630 nm laser, 250 J/cm^2^ for 25 min; plastic stent or drainage catheter; follow-up every 3 months	Median survival favored PDT + stent over stent-only treatment (14.2 vs. 9.8 months; *p* = 0.003)	Reported bilirubin and QoL improvement; postoperative adverse events were not significantly different between groups	Single-center observational design
Möhring et al., 2023 [20]	Prospective comparative; *n* = 63	Advanced extrahepatic biliary obstruction	Endobiliary RFA comparator; PDT/RFA delivered with systemic-therapy context; PDT delivery details were incompletely or variably reported; median follow-up of 13.5 months	No stable comparative survival advantage established	Comparative patency and safety interpretation limited by protocol heterogeneity and incomplete delivery reporting	Anatomy and delivery reporting were incomplete
Mohan et al., 2021 [12]	Systematic review/meta-analysis	Unresectable extrahepatic CCA; mixed endobiliary studies	Stent-based comparators; PDT/RFA studies with study-level follow-up variation	Survival signal mixed across included studies	Some pooled analyses favored local ablation for biliary outcomes, with substantial heterogeneity	Dependent on older and observational source studies
Yu et al., 2023 [24]	Systematic review/meta-analysis	Unresectable extrahepatic CCA; PDT plus systemic therapy studies	Systemic therapy context emphasized; study-level follow-up varied	Pooled survival signal favored PDT + chemo	Supports treatment-context relevance, not proof of independent PDT survival benefit	Predominantly nonrandomized heterogeneous literature

Table notes: PFS, progression-free survival; QoL, quality of life; BTC, biliary tract cancer; RFA, radiofrequency ablation; ERCP, endoscopic retrograde cholangiopancreatography; NR, not reported or not consistently extractable from the available source. Survival signal is a qualitative summary rather than a pooled effect estimate. Where detailed PDT parameters were clearly available, they are summarized in the comparator/details column; otherwise, delivery details described as variably reported or NR. Across studies, endobiliary PDT evidence is concentrated in obstruction-predominant extrahepatic or perihilar disease, and mixed cohorts should not be uncritically extrapolated to mass-forming iCCA. The main outcome domains used to interpret endobiliary PDT studies are summarized in Table 4.

**Table 4:** Outcome domains for clinical interpretation of endobiliary PDT studies.

Outcome Domain	Most Consistent Signal	What Limits Confidence	Clinical Reading
Overall survival (OS)	Early positive studies and later observational cohorts conflict with negative randomized evidence.	Small samples, nonrandomized designs, metastatic burden, drainage and infection confounding, downstream systemic therapy.	Should not interpret PDT as an established survival-prolonging treatment.
Biliary decompression/stent dysfunction	More consistently favorable in selected obstruction-predominant extrahepatic or perihilar disease.	Variable endpoint definitions, retreatment rules, and stent strategies.	Currently, this is the strongest rationale for selected local biliary palliation in obstruction-predominant extrahepatic or perihilar disease.
Quality of life (QoL)/symptom burden	Some studies report benefit, but validated QoL data are limited.	Sparse prespecified QoL collection and inconsistent timing.	Potential patient-centered benefit should be weighed against repeat intervention burden.
Cholangitis/infectious complications	Clinically important competing morbidity, but frequency varies across studies.	Heterogeneous definitions, drainage adequacy, antibiotic strategy, and event-attribution windows.	Interpret as a major safety and confounding issue rather than as a PDT-specific effect in isolation.
Repeat intervention/procedure burden	Additional ERCP, reassessment, or retreatment may be required in some PDT treatment pathways.	Variable retreatment rules, stent-exchange practices, follow-up intensities, and center workflows.	Consider as a context-dependent tradeoff that may offset biliary benefit in some patients or settings.

Taken together, current studies suggest that endobiliary PDT is best understood as a context-dependent local biliary palliative strategy whose clinical signal is driven more reliably by biliary than by oncologic endpoints. Survival benefit remains uncertain, and practical value depends in part on whether local palliation appears to improve decompression, infection control, retreatment burden, and continuity of systemic therapy in an individual patient.

In this review, randomized and prospective comparative studies are treated as the most informative clinical evidence, with observational cohorts used for context and mechanistic data treated as hypothesis-generating rather than practice-defining.

### 3.5. Future Research Priorities

Table 5 summarizes the variables most likely to improve interpretability are anatomy-aware enrollment, explicit drainage targets, protocolized infection management, defined retreatment rules, reproducible light-delivery records, and clear documentation of systemic-therapy timing. These elements do not guarantee efficacy, but they help readers judge whether survival or biliary signals are being attributed to comparable clinical situations. Registered biliary PDT trials are summarized in Supplementary Table S1.

### 3.6. Practical Implications and Low-Yield Settings

For unresectable CCA, endobiliary PDT is used in a palliative setting in which feasibility, adverse events, and healthcare use are part of the clinical signal rather than peripheral implementation details [12,13]. Cholangitis, stent dysfunction, photosensitivity management, repeat ERCP, and access to photosensitizers or compatible equipment all influence whether a local biliary strategy remains worthwhile [11,18,25,33,34].

### 3.7. Clinical Boundaries and Low-Yield Settings

Even with careful procedural reporting, biliary PDT is unlikely to be equally persuasive across all cholangiocarcinoma contexts. The clearest use case remains nonresectable extrahepatic or perihilar obstruction where maintaining ductal patency, bilirubin control, and continuity of systemic therapy are meaningful objectives in their own right. The rationale weakens substantially when trajectories are dominated by rapidly progressive metastatic disease, profound frailty or hepatic failure, uncontrolled infection, inability to achieve a prespecified drainage target, or predominantly mass-forming intrahepatic disease that is poorly represented in endobiliary datasets.

These settings are better regarded as low-yield or difficult-to-interpret contexts unless a study provides a specific rationale for inclusion. Counterevidence should therefore function as a boundary-setting signal, not merely as heterogeneity around a positive narrative.

## 4. Outcome Measures for Future Studies

OS remains clinically important but is particularly vulnerable to confounding in this literature. Future studies would be easier to interpret if OS were reported alongside:

- Time to stent dysfunction or reintervention;
- Bilirubin response and durability;
- Cholangitis defined by TG18 criteria;
- Hospitalization burden;
- Validated QoL instruments (e.g., EORTC QLQ-BIL21);
- Ability to deliver intended systemic therapy without biliary interruption.

These composite endpoints would better capture the clinical value of local biliary palliation [10,11,12,13,18,25,31,32].

These outcome layers depend on variables that are partly controllable and, at minimum, documentable: drainage adequacy, infection control, treated-segment coverage, light-delivery geometry, photosensitizer exposure, and retreatment policy. The practical task is to record enough procedural context to make study results clinically readable rather than to imply that reporting alone can resolve efficacy uncertainty.

Negative and positive studies should therefore be read as signals within a procedure-sensitive clinical context, not as simple proof for or against PDT in all cholangiocarcinoma settings.

### Condensed Procedural Context

Technical detail remains relevant only insofar as it changes clinical interpretation. The most portable descriptors are treated segment, diffuser length, power or power per diffuser length, treatment time, resulting energy per diffuser length, drug-light interval, catheter positioning method, and whether overlapping illuminations or repeat sessions were used [1,2,14,27,39].

Studies that omit these elements are harder to compare, but detailed dosimetry discussion should remain subordinate to clinical outcomes in a narrative review. More extensive technical material has therefore been kept in the supplement [1,14,17,18,19]. Additional reporting items and technical details are summarized in Supplementary Tables S3 and S4.

## 5. Exploratory Translational Directions

**Figure 2:**
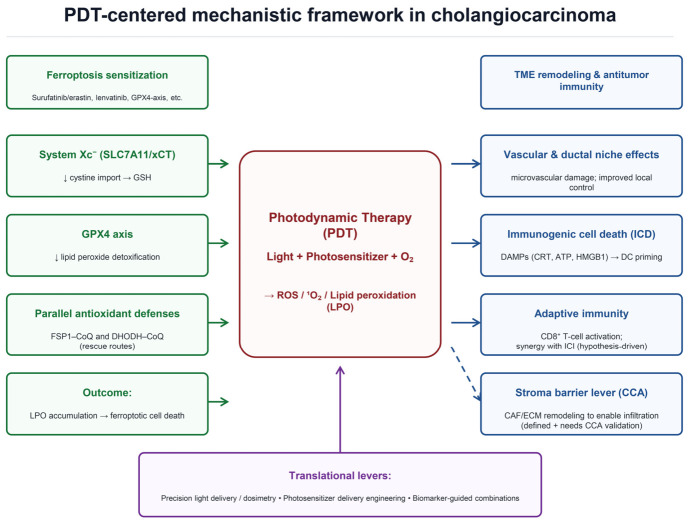
Exploratory mechanisms relevant to PDT intensification in cholangiocarcinoma. The figure summarizes predominantly preclinical or indirect evidence linking PDT-induced reactive oxygen species (ROS), lipid peroxidation (LPO), ferroptosis sensitization, tumor microenvironment remodeling, immunogenic cell death, adaptive immunity, and translational levers such as light delivery, photosensitizer delivery, and biomarker-guided combinations. This framework is hypothesis-generating and should not be interpreted as a clinically validated map of human endobiliary PDT response.

Exploratory combination strategies should not be presented as a way to rescue an uncertain local procedure. Figure 2 summarizes largely preclinical concepts involving ferroptosis, immune modulation, targeted therapy, and delivery engineering; these ideas remain hypothesis-generating and require human biliary validation before they can influence routine clinical interpretation [36,42,43,44,45,46,47,48,49,50,51,52,53,54,55,56,57,58,59,60,61,62,63,64,65,66,67,68,69,70,71,72,73,74,75,76,77,78]. Additional mechanistic and translational evidence is summarized in Supplementary Table S2.

In the same spirit, biomarkers such as bile or brushing correlates, circulating tumor DNA (ctDNA), inflammatory markers, or oxidized lipid signatures may be informative only if linked to sampling timing, drainage status, cholangitis, antibiotic exposure, and procedural metadata. At present, they should not be treated as validated response markers for endobiliary PDT [37,51,57,65,66,67,72,73,74,75].

## 6. Conclusions

Therefore, the current evidence supports endobiliary PDT as a context-dependent palliative biliary intervention in selected obstruction-predominant extrahepatic or perihilar disease, but does not support broad claims of survival prolongation across unselected CCA populations.

This narrative review treats procedural context as essential to interpreting outcomes, not as a substitute for outcome evidence itself. Future studies will be most useful when they report anatomy, drainage targets, stent and antibiotic strategy, photosensitizer and light-delivery parameters, retreatment rules, and patient-centered as well as biliary endpoints. Translational combinations and biomarker strategies remain exploratory until validated in human biliary PDT cohorts. Priority areas for future research include standardized dosimetry protocols, prospectively collected biomarker candidates linked to procedural metadata, and trials evaluating PDT within contemporary systemic-therapy backbones in well-defined anatomic and disease-stage subgroups.

## Figures and Tables

**Table 1:** Key take-home messages for clinical interpretation of endobiliary PDT.

Domain	Take-Home Message
Clinical role	Endobiliary PDT is best supported as selected local palliation for obstruction-predominant extrahepatic or perihilar disease.
Survival	Overall survival benefit is inconsistent and should be interpreted alongside drainage adequacy, cholangitis, retreatment burden, and systemic-therapy delivery.
Reporting priorities	Comparative studies are most interpretable when they describe anatomy, drainage targets, stent and antibiotic strategy, photosensitizer type/dose/interval, light-delivery parameters, and retreatment rules.
Translational status	Ferroptosis-based, targeted, immune, and biomarker strategies remain exploratory until validated in human biliary PDT cohorts.
Resource implications	Procedure burden, photosensitizer logistics, repeat ERCP, and equipment availability are part of the clinical context rather than secondary implementation details.

**Table 5:** Clinical and reporting variables that most affect interpretation of endobiliary PDT studies.

Variable (Report/Stratify)	Why It Modifies Efficacy/Safety	Reporting/Design Priority
Disease context (stage; metastatic vs. non-metastatic; iCCA vs. pCCA/dCCA; Bismuth type for hilar)	Determines whether local ductal control can plausibly influence outcomes; anatomy drives coverage feasibility and infection risk.	Pre-specify eligibility/subgroups; do not pool iCCA with extrahepatic; report stricture extent/branch involvement; stratify by metastatic status and hilar anatomy.
Drainage adequacy & stent strategy (drained liver volume; unilateral/bilateral; type; exchange policy)	Incomplete drainage and undrained contrast-opacified ducts increase cholangitis and can dominate morbidity/endpoints.	Define drainage goals (planned segments/volume), stent plan, and reintervention triggers; harmonize across arms in comparative studies.
Systemic-therapy backbone & timing relative to PDT	OS and even durability endpoints can be confounded by access to/continuity of chemotherapy and supportive care.	Specify allowable regimens and timing windows; document delays/omissions; consider stratifying by systemic-therapy receipt.
Light delivery reproducibility (diffuser length, centering/position verification, overlap/pullback strategy, calibration)	Positioning and device differences can cause under- or over-delivery, manifesting as nonresponse or biliary toxicity.	Predefine coverage targets and segment strategy; record diffuser active length, mW/cm, J/cm, time, calibration method; document positioning verification.
Infection prevention/adjudication (antibiotics; TG18 cholangitis definition; timing windows)	Biliary infection is a dominant competing risk and confounder for safety and efficacy signals; using TG18-based criteria can standardize attribution through systemic inflammation, cholestasis, and imaging evidence.	Protocolize antibiotic approach when incomplete drainage anticipated; use TG18-based definitions; predefine attribution windows for post-PDT events.
Repeat-PDT policy (triggers, reassessment interval)	Retreatment frequency changes cumulative exposure and affects patency and resource-use endpoints.	Predefine reassessment schedule and retreatment triggers; record number and timing of repeat sessions.

Abbreviations: iCCA, intrahepatic cholangiocarcinoma; pCCA, perihilar cholangiocarcinoma; dCCA, distal cholangiocarcinoma; OS, overall survival; TG18, Tokyo Guidelines 2018.

## Data Availability

No new datasets were generated or analyzed for this review. All information discussed in this article is derived from published sources, which are cited in the reference list.

## Supplementary Materials

The following supporting information can be downloaded at https://www.mdpi.com/article/10.3390/curroncol33060343/s1, Supplementary Table S1 (registered biliary PDT trials), Supplementary Table S2 (mechanistic and translational evidence), Supplementary Table S3 (manuscript reporting items), and Supplementary Table S4 (technical details for endobiliary PDT reports).
