# Supplementary material for: Endobiliary Photodynamic Therapy in Cholangiocarcinoma: Clinical Outcomes, Patient Selection, and Procedural Context
Source: Curr Oncol. 2026 Jun 9;33(6):343. doi: 10.3390/curroncol33060343 (PMC13298456; doi:10.3390/curroncol33060343)
Supplement: Supplementary file 1 [file curroncol-33-00343-s001.zip › curroncol-4282833-supplementary.pdf]

Supplementary Materials

Supplementary Table S1

Supplementary Table S1. Registered biliary PDT trials: design features relevant to interpretability.

Trial status is subject to change. This table reflects registry information available as of 31 December 2025; records not updated for more than 5 years may be inactive or discontinued.

Registry ID	Phase / Design	**Intervention**	**Population**	Primary endpoint(s)	**Status**
NCT 02082522	Phase III; randomized	Porfimer sodium PDT + SMC vs SMC	Advanced/unresectable CCA	Overall survival; safety	Terminated early (Enrollment 28; primary completion 2017-01-12; record last updated 2019-08-28). Note: Early termination due to slow enrollment; no registry results were posted as of 31 December 2025.
NCT 01739465	Randomized; comparative	Endobiliary PDT vs endobiliary RFA	Inoperable CCA	Survival; stent patency; AEs	Unknown (record last updated 2013-02-25; verify current status)
NCT 05519319	Randomized	PDT vs RFA (with stenting)	Unresectable extrahepatic CCA	OS; stent patency; AEs	Recruiting (record last updated 2023-02-18)
NCT 00869635	Phase II; randomized	PDT + S-1 vs PDT alone	Unresectable hilar CCA	Survival; stent patency	Completed (record last updated 2014-02-06)
NCT 04824742	Neoadjuvant; randomized	Neoadjuvant PDT protocol + surgery vs comparator	Locally advanced CCA planned for resection	R0 resection; recurrence; safety	Not yet recruiting (record last updated 2021-03-30)
NCT 02725073	Registration record	PDT in locally advanced hilar CCA	Hilar CCA	Feasibility / clinical outcomes	Unknown (site listed as recruiting; record last updated 2016-04-12)

Supplementary Table S2

Supplementary Table S2. Mechanistic and translational evidence informing PDT-based combinations, stratified by evidence level.

This table separates CCA-anchored observations (n=3: Huang 2024, Zhang 2021, Dong 2024) from broader PDT biology (n=2: Shui 2021, Sorrin 2020) and methodological or delivery advances (n=3: Li 2024, Wang 2022, selected reviews). All entries should be interpreted as hypothesis-generating rather than practice-defining.

**Study**	**Model**	**Combination/concept**	**Key mechanistic claim**	**Key assays**	**DOI / identifier**
Shui et al., 2021 [37]	Multiple cancers (mechanistic anchor)	PDT→non-enzymatic LPO	PDT initiates ferroptosis-like death via non-enzymatic LPO	LPO; lipidomics; rescue	10.1016/j.redox.2021.102056
Huang et al., 2024 [56]	CCA in vitro + tumor models	Surufatinib + PDT	Synergy via ferroptosis programs	LPO; GPX4; ACSL4; inhibitors	10.3389/fphar.2024.1288255
Zhang et al., 2021 [47]	CCA (HuCC-T1) models	Mesoporous silica platform	Improved delivery/efficacy in models	Uptake; ROS; tumor growth	10.3389/fonc.2021.665182 (PMC8276239)
Sorrin et al., 2020 [38]	Review	TME biophysics in PDT	PDT can be engineered to target cellular & non-cellular TME components	Conceptual framework	10.3390/cancers12040934
Li et al., 2024 [48]	Review	Smart nanotechnology-supported PDT	Nanotechnology enhances delivery & reduces toxicity	Conceptual framework	10.1038/s41420-024-02236-4
Wang et al., 2022 [41]	Method paper	Monte Carlo TPS (FullMonteWeb)	Accessible simulation for plan optimization	Light propagation simulation	10.1117/1.JBO.27.8.083006
Dong et al., 2024 [57]	CCA (QBC939) in vitro	Hematoporphyrin-PDT + ferroptosis sensitization (erastin or lenvatinib); Fer-1 rescue	PDT engages ferroptosis-associated redox programs; erastin/lenvatinib amplify ROS/LPO and suppress GPX4/FSP1/SLC7A11; Fer-1 attenuates effects	ROS; GSH; Fe^{2+}; LPO/MDA; Western blot (GPX4, FSP1, SLC7A11, ACSL4); viability; apoptosis/cell cycle; Fer-1 rescue	10.1002/lsm.23857

Supplementary Table S3

Supplementary Table S3. Reporting items for biliary PDT manuscripts.

This table summarizes reporting items that may help reduce avoidable heterogeneity in future biliary PDT manuscripts.

**Category**	**Items to report (minimal core)**
Photosensitizer (PS)	Name/class; dose; route; drug–light interval; contraindications; hepatic/biliary clearance
Light source	Wavelength, power at fiber tip; calibration method; device model
Dosimetry	Report the core procedure-level dosimetry dataset, including power output, illumination time, energy per diffuser length, treated length, overlap or pullback strategy, and relevant assumptions. Add coverage rationale (treated length, overlap/pullback plan) and, if estimating duct-wall fluence/irradiance, state the method and assumptions.
Delivery/access	ERCP vs PTCS; fiber type; position confirmation; number of PDT sessions and interval
Biliary intervention	Stent type/number; drainage adequacy; antibiotics; cholangitis prophylaxis/management
Clinical context	Anatomic subtype; bilirubin/ALP/CRP; ECOG; systemic therapy timing; obstruction severity
Tumor biology	Hypoxia indicators; stromal markers (CAF/ECM); baseline immune contexture
Mechanistic readouts	ROS/LPO; 4-HNE/MDA; Fe^{2+}; GPX4/SLC7A11/FSP1/DHODH; rescue controls (Fer-1/Lip-1 and/or DFO)
Outcomes	Stent patency; bilirubin response; QoL; adverse events; OS/PFS when applicable

Supplementary Table S4

Supplementary Table S4. Technical details for endobiliary PDT reports.

**Domain**	**Minimum items to report**	**Practical notes/rationale**
Photosensitizer	Agent; dose; infusion method; drug-light interval; photosensitivity precautions provided	These variables modulate intraductal drug exposure and the safety counseling burden.
Light source	Wavelength; device/manufacturer; calibration method; output stability checks	Wavelength must match photosensitizer absorption; calibration reduces between-center drift.
Fiber and catheter	Diffuser type (cylindrical vs point); diffuser active length; catheter size; guidance modality (fluoroscopy vs cholangioscopy)	Active length should cover the stricture with defined overlap; cholangioscopy may improve targeting in complex hilar anatomy.
Light delivery plan	Power output (total in mW and per diffuser length in mW/cm); light delivery time (seconds or minutes, specify); energy per diffuser length (J/cm, calculated as power × time ÷ length); number of light delivery positions/pullbacks; planned overlap length; and, if	Report per-segment parameters because long strictures are frequently treated in staged segments. Unit conventions: use consistent units across reports (mW rather than W where possible; seconds or minutes explicitly specified; J/cm rather than J/cm² for cylindrical diffusers).

Domain	Minimum items to report	Practical notes/rationale
	reported, estimated duct-wall fluence/irradiance with stated assumptions.	
Biliary drainage context	Stricture location (Bismuth type if hilar); stent type/number; drainage adequacy criteria before PDT	Drainage adequacy and stent strategy are major effect modifiers for cholangitis risk and subsequent systemic therapy delivery.
Infection prophylaxis	Antibiotic regimen; cholangitis definition/grade; culture strategy if febrile	Standardized definitions (e.g., TG18) and prophylaxis reporting improve AE comparability.
Follow-up and retreatment	Planned surveillance schedule; triggers for re-intervention (biochemical relapse, stent dysfunction, cholangitis); repeat PDT policy.	Explicit triggers reduce bias in stent patency and re-intervention endpoints.
